# *In vivo* Pharmacokinetic/Pharmacodynamic (PK/PD) Profiles of Tulathromycin in an Experimental Intraperitoneal *Haemophilus parasuis* Infection Model in Neutropenic Guinea Pigs

**DOI:** 10.3389/fvets.2021.715887

**Published:** 2021-11-12

**Authors:** Li-li Guo, Rui-yuan Gao, Li-hua Wang, Shu-jun Lin, Bing-hu Fang, Yong-da Zhao

**Affiliations:** ^1^College of Veterinary Medicine, Qingdao Agricultural University, Qingdao, China; ^2^National Risk Assessment Laboratory for Antimicrobial Resistance of Microorganisms in Animals, College of Veterinary Medicine, South China Agricultural University, Guangzhou, China

**Keywords:** PK/PD, tulathromycin, *in vivo*, *Haemophilus parasuis*, guinea pig

## Abstract

Tulathromycin is a semi-synthetic macrolide antimicrobial that has an important role in veterinary medicine for respiratory disease. The objective of the study was to develop a pharmacokinetic/pharmacodynamic (PK/PD) model to examine the efficacy and determine an optimal dosage of tulathromycin intramuscular (IM) treatment against *Haemophilus parasuis* infection induced after intraperitoneal inoculation in neutropenic guinea pigs. The PKs of tulathromycin in serum and lung tissue after intramuscular administration at doses of 1, 10, and 20 mg/kg in *H. parasuis*-infected neutropenic guinea pigs were evaluated by liquid chromatography-tandem mass spectrometry (LC-MS/MS). The tulathromycin minimum inhibitory concentration (MIC) against *H. parasuis* was ~16 times lower in guinea pig serum (0.03 μg/mL) than in cation-adjusted Mueller-Hinton broth (CAMHB) (0.5 μg/mL). The ratio of the 168-h area under the concentration-time curve (AUC) to MIC (AUC_168h_/MIC) positively correlated with the *in vivo* antibacterial effectiveness of tulathromycin (*R*^2^ = 0.9878 in serum and *R*^2^ = 0.9911 in lung tissue). The computed doses to achieve a reduction of 2-log_10_ CFU/lung from the ratios of AUC_72h_/MIC were 5.7 mg/kg for serum and 2.5 mg/kg for lung tissue, which lower than the values of 13.2 mg/kg for serum and 8.9 mg/kg for lung tissue with AUC_168h_/MIC. In addition, using as objective a 2-log_10_ reduction and an AUC_0−72h_ as the value of the PK/PD index could be more realistic. The results of this study could provide a solid foundation for the application of PK/PD models in research on macrolide antibiotics used to treat respiratory diseases.

## Introduction

*Haemophilus parasuis*, a Gram-negative bacterium (1), is a commensal organism of the upper respiratory tract and a major cause of swine respiratory disease (SRD) in swine. It is also the etiological agent of Glasser's disease, the clinical symptoms of which are high fever, severe coughing, abdominal breathing, and swollen joints, in addition to the central nervous system (CNS) signs such as lateral decubitus, paddling, and trembling (2–5). At least 15 different genotypes and serotypes of *H. parasuis* have been described (6, 7) where serotypes 4, 5, and 13 are the most prevalent in some countries of America, Europe, and Asia (8–17) and serotypes 5 and 13 are the most virulent of all the known serotypes (18, 19).

Usually, the relationship of pharmacokinetics (PK) and pharmacodynamics (PD) is used to design the optimal antimicrobial drug dosage (20–23), and to minimize the risk of selection of antibiotic resistance (20, 24, 25). Thus, PK/PD modeling has become an important tool and has been widely applied in human medicine and the veterinary field (26–28). In particular, an *in vivo* PK/PD model has some advantages over an *ex vivo* PK/PD model by avoiding some of the limitations of *ex vivo* PK/PK models. For example, in drug investigation of drug metabolism and body clearance, *ex vivo* PK/PD models do not exhibit the natural decline in concentration of antibiotics through drug elimination in animals because the animals are continuously exposed to a fixed concentration of the agent over a prescribed period of time (29). In addition, the concentration of a drug in the target site usually varies from that in serum or tissue cage fluid. Because *in vivo* PK/PD models can account for natural changes in antibacterial concentrations, they can provide more accurate clinical data (29).

Tulathromycin is a semi-synthetic macrolide developed solely for veterinary use by the pharmaceutical company Pfizer Inc. for its potent activity against *H. parasuis* (30, 31). In veterinary medicine, tulathromycin is highly effective against respiratory diseases in cattle, swine, horses, and goats (32–37). Recently, researchers used serum or tissue cage fluid to investigate the pharmacokinetics and *ex vivo* pharmacodynamic activity of tulathromycin against *Pasteurella multocida* (38) and *Streptococcus suis* (39) in pigs, and against *Mannheimia haemolytica* and *Pasteurella multocida* (40) in cattle, and *ex-vivo* PK/PD model of tulathromycin against *H. parasuis* in intraperitoneal-inoculated neutropenic guinea pigs (41). However, no study has reported on *in vivo* PK/PD profiles of tulathromycin in pigs.

Currently, many PK attributes of tulathromycin have been studied in different target animals, such as goats, horses, pigs, and cattle (32, 42–46). However, of the common laboratory animals, only mice (47) and rabbits (48) have been used in tulathromycin studies, whereas, guinea pigs have not. Nevertheless, many reports have shown that guinea pigs can be used as pneumonia models for Gram-negative bacilli (49). Furthermore, guinea pigs have been used as an effective animal model to study the pathogenesis and diagnosis of and immunization against *H. parasuis* infection (18, 50, 51). Thus, we endeavored to examine the PK/PD of a commonly used antibiotic macrolide, tulathromycin, in a guinea pig infection model.

An experimental *H. parasuis*-infected, neutropenic guinea pig model was developed to investigate the PKs and PDs of tulathromycin and its efficacy against infections *in vivo*. The quantity of *H. parasuis* in serum and lung tissue samples was detected using the viable count method. Our *in vivo* PK/PD study had two primary objectives: (1) to determine a PK/PD index associated with the potential variability of tulathromycin efficacy; (2) to elucidate the optimal PK/PD parameters to predict efficacy; (3) to assess the different possible clinical situations ranging from a mild infection in a non-immunocompromised animal up to a severe infection in an immunocompromised animal. The data from this study can help improve the use of tulathromycin treatment with respect to bacteriological and clinical outcomes by providing a rational approach to the design of optimal dosage regimens for target animals.

## Materials and Methods

### Organisms, Chemicals, and Susceptibility Assay

A standard strain of *H. parasuis*, serotype 13 (13R), was provided by Professor Huanchun Chen (College of Veterinary Medicine, Huazhong Agricultural University, Wuhan, China), which is one of the most prevalent and the most virulent of *H. parasuis* (8–13, 16, 17, 19). Tulathromycin (99.85%) was supplied by Shandong Lukang Pharmaceutical Co., Ltd (Shandong, China). Roxithromycin was purchased from National Institutes for Food and Drug Control (Beijing, China) and used as an internal standard. An injectable solution of tulathromycin (Draxxin) was purchased from Zoetis (New York, USA) and used for IM administration to guinea pigs. The MIC of tulathromycin against *H. parasuis* 13R was determined by a microdouble-dilution method according to protocols by the Clinical and Laboratory Standards Institute (52) and a previous report (53). The *H. parasuis* strain was cultured to reach the exponential phase and diluted to ~1 × 10^6^ CFU/mL. A series of concentrations of tulathromycin were prepared by doubling dilutions (final concentrations ranged from 0.015 to 8 μg/mL) with CAMHB medium. A 0.1 mL aliquot of this media with different concentrations of tulathromycin was added to a 96-well plate and another 0.1 mL aliquot of prepared *H. parasuis* 13R was added to each well to reach final titers of ~5 × 10^5^ CFU/mL. Tests were conducted in triplicate and included growth controls (*H. parasuis* in the CAMHB only), and germ-free controls (blank media only). The bacteria in the 96-well plates were cultured at 37°C for 24 h. The MIC value was defined as the lowest concentration exhibiting no visible growth of *H. parasuis*. The MIC of tulathromycin against *H. parasuis* 13R in serum was determined by the same method described above.

### Experimental Models and Sample Collection

#### Neutropenia Model

In order to study the efficacy of tulathromycin, alone, and to reduce the inherent variability in immunity among different guinea pigs, a neutropenic model was used. The Qingdao Agriculture University Animal ethics committee approved the animal experiment procedures (No. 2020-025). All procedures were performed to minimize animal suffering as defined by the guidelines issued by this committee. Three-week old guinea pigs (240–250 g) were provided by the Laboratory Animal Center, Qingdao Agriculture University in Qingdao, China (License number: SCXK (LU) 2018–2022). Guinea pigs were free of *H. parasuis* and fed antibacterial-free food and water *ad libitum*. Three days post arrival, guinea pigs were rendered neutropenic by intraperitoneal injection (IP) with cyclophosphamide (TCI (Shanghai) Development Co., Ltd, Shanghai, China) at 100 mg/kg one time a day for 3 days. Blood samples were drawn from the heart.

#### *H. parasuis* Intraperitoneal Infection Model

Neutropenic guinea pigs were inoculated with a 0.2 mL aliquot solution containing ~1 × 10^9^ CFU of the *H. parasuis* strain and was equivalent to a 95% infective dose (ID_95_) as determined in pilot studies on intraperitoneal injection for single-dose. Clinical symptoms were recorded and bacteriological examinations were performed to ensure infection. To quantify pathogen load, lung tissue was removed at sacrifice by using an overdose of pentobarbital.

### Tulathromycin PKs in the Neutropenic Intraperitoneal Infection Model

A total of 384 infected, neutropenic guinea pigs were allocated to three groups and treated with tulathromycin at single IM doses of 1, 10, or 20 mg/kg, respectively. Aliquots of 2 mL blood samples were collected from the heart at 5, 10, 15, and 30 min, and 1, 2, 4, 8, 12, 24, 48, 72, 96, 120, 144, and 168 h after drug administration. Eight guinea pigs were sampled at each time point. All experimental animals were euthanized by using an overdose of pentobarbital after blood sampling. Lung tissue samples were homogenized and immediately placed in cryo-tubes to freeze at −20°C. Serum was obtained by centrifuging blood samples at 4,000 g for 10 min and immediately stored at −20°C until analysis (within 1 month of sampling).

### Analysis Methods

#### Neutropenia Model

Leukocytes were counted with an automatic blood cell analyzer (Mindray BC-2800Vet, Shenzhen, China). Animals were severely granulocytopenic (absolute leukocyte count <1,000/mm^3^).

#### *H. parasuis* Intraperitoneal Infection Model

Lung tissue was homogenized in 0.5 mL of phosphate-buffered saline (PBS), and centrifuged at 7,200 *g* for 15 s by a Precellys Evolution Super Homogenizer (Bertin Technologies, France). Aliquots of 0.1 mL of the supernatant were used in 10-fold serial dilutions that were incubated at 37°C for 36–48 h. Subsequently, viable counts were obtained by the spot-plate method. Blood samples were collected from the heart before euthanasia and obtaining viable counts. The lowest detectable count was 100 CFU/mL. All samples were performed in triplicate.

#### Tulathromycin PKs

Tulathromycin concentrations in serum and lung were determined *via* a high-performance LC-MS/MS method as described previously (54, 55). The limit of quantitation was confirmed at 2.0 ng/mL for serum and 10 ng/g for lung tissue. The coefficient of correlation (*r*^2^) was 0.9974 for the linear range of 2.0–500 ng/mL for serum and 0.9941 for the linear range of 10–1,000 ng/mL for lung tissue. To overcome the carryover effect, water was inserted in the detection queue after every 10 samples. Over the validated range, plasma assay accuracy and precision were in the ranges of 94.6–101.5 and 2.2-−6.8%, respectively. For lung tissue, the assay accuracy and precision were in the ranges of 89.4–98.2 and 5.6–12.4%, respectively.

### Efficacy of Tulathromycin in the Neutripenic Guinea Pig Intraperitoneal Infection Model

To evaluate the effectiveness of tulathromycin, infected, neutropenic guinea pigs were treated with either 0.9% NaCl (Control group) or tulathromycin at 1, 2, 4, 6, 8, 10, 15, or 20 mg/kg (Treatment group), at 2 h post-infection of a single dose. Three days after IM drug administration, animals were euthanized after blood sampling. Then the lungs were collected, homogenized in 0.5 mL PBS, and centrifuged at 7,200 rpm for 15 s. The viable counts of *H. parasuis* were measured by the viable count method as described above. The number of *H. parasuis* was expressed as CFU/mL.

### PKs and PDs Analyses

The PK profiles of tulathromycin were analyzed by the non-compartmental model with uniform weighting using WinNonlin software (version 5.2; Pharsight, CA, USA). The surrogate marker of antibacterial effectiveness, AUC_168h_/MIC, was calculated using *in vitro* MIC values and PK parameters obtained from lung tissue samples from three individuals treated with IM administrations of tulathromycin. The effectiveness of tulathromycin was expressed as a reduction of *H. parasuis* loading calculated by subtracting loading of the treatment group from the control group. The *in vivo* PK/PD relationship of tulathromycin was described using a sigmoid inhibitory E_max_ model with the WinNonlin software (version 5.2; Pharsight, CA, USA) where the equation is as follows:


$$\begin{array}{l} E=E_{\max}-\left(E_{\max}-E_{0}\right)\times \frac{C_{e}^{N}}{EC_{50}^{N}+C_{e}^{N}} \end{array}$$

where *E* is the antibacterial effect measured as the reduction in log_10_ CFU/lung after administration of tulathromycin compared to that of the log_10_ CFU/lung in the untreated control group; *E*_*max*_ is the reduction in log_10_ CFU/lung for the untreated control guinea pigs; *E*_0_ is the maximum reduction after administration and represents the maximum antibacterial effect; *Ce* is the AUC/MIC_serum_ parameter; *EC*_50_ is the AUC/MIC_serum_ value required to achieve 50% of the maximal antibacterial effect; and *N* is the Hill coefficient that describes the steepness of the AUC/MIC_serum_ and effect curve.

### Dosage Calculation

In order to determine a more optimal dose regimen for treatment of *H. parasuis*, the dose required for a given antibacterial activity is calculated by the equation (56):


$$\begin{array}{l} Dose_{\left(for\;5\;days\right)}=\frac{Cl_{\left(for\;5\;days\right)}\times factor\times MIC_{90}}{fu\times F} \end{array}$$

where Dose is the optimal dose (mg/kg/day); Cl is the serum (or lung) clearance; factor is the dimensionless numerical value of AUC/MIC_serum_; MIC_90_ is the 90th percentile of the MIC distribution; *fu* is the free drug fraction; and F is the bioavailability.

## Results

### Susceptibility Testing

The MICs of tulathromycin against the study strain in CAMHB and serum were 0.5 and 0.03 μg/mL, respectively.

### Neutropenia Model

Animals were severely granulocytopenic (absolute leukocyte count <1,000/mm^3^), and remained in this condition for at least 3 days after the last injection of cyclophosphamide.

### *H. parasuis* Intraperitoneal Infection Model

Evaluation of the *H. parasuis* infection model mainly depended on clinical symptoms and bacteriological assays. Depression, mouth breathing, and roughened hair coats were observed in infected animals. In addition, infection was observed in 100% of the inoculated guinea pigs. Mean *H. parasuis* load in lungs was 4.5 × 10^6^ CFU/g and in blood was 1 × 10^7^ CFU/mL for all inoculated guinea pigs. Morbidity and mortality rates were 100 and 15% within 3 days after infection, respectively. Neither clinical sign was observed in non-infected control animals. The bacteriological assay was negative in control guinea pigs.

### PK Profiles of Tulathromycin in the Neutropenic Intraperitoneal Infection Model

**Figure 1:**
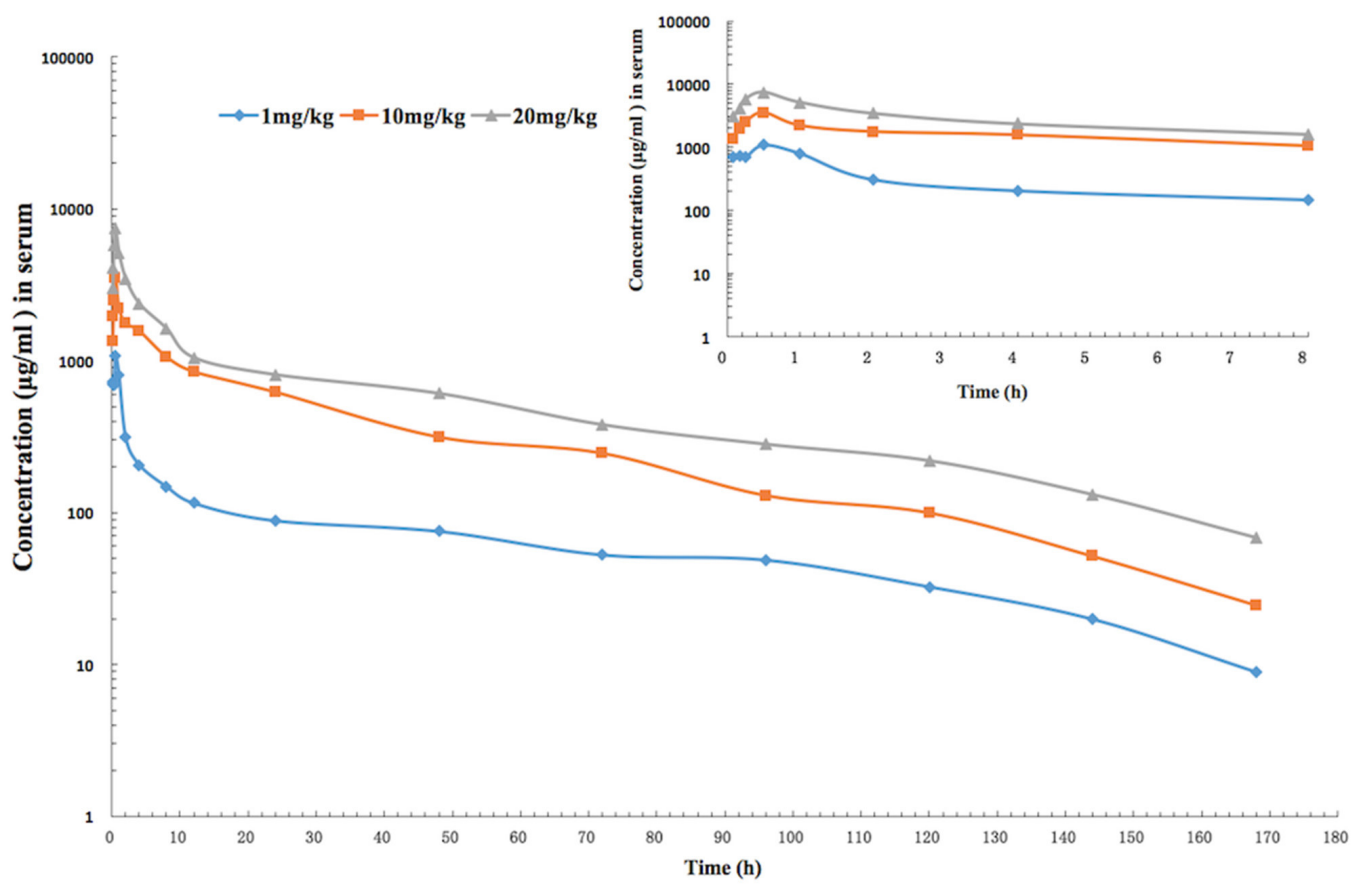
The time-concentration curve of tulathromycin in serum for each of three intramuscular administrations of 1, 10, and 20 mg/kg in a *H. parasuis* infection model (inset depicts the serum concentrations in the first 8 h post-administration; *n* = 8/time point).

**Figure 2:**
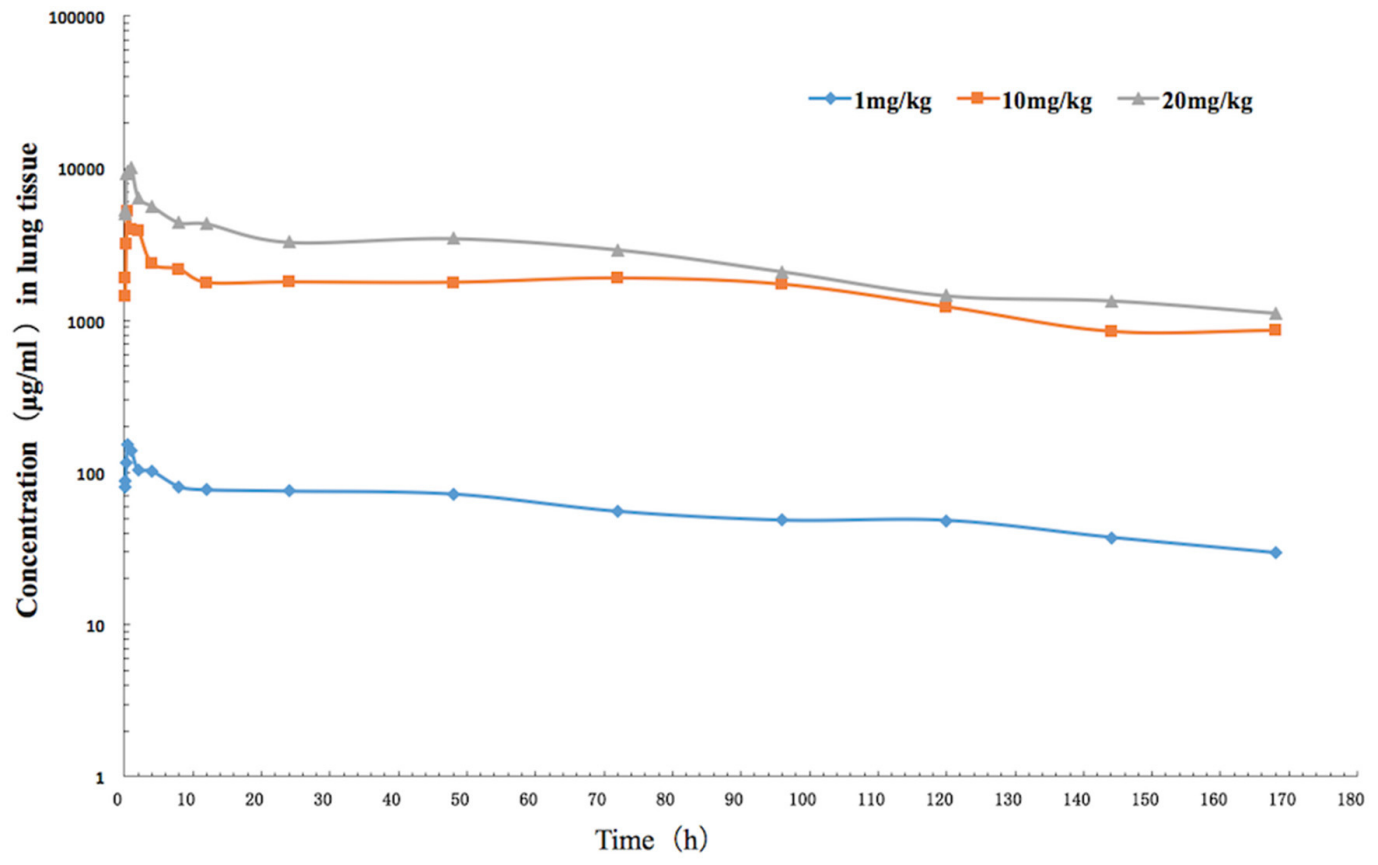
The time-concentration curve of tulathromycin in lung tissue for each of three intramuscular administrations of 1, 10, and 20 mg/kg in a *H. parasuis* infection model (*n* = 8/time point).

The mean values of PK parameters of serum samples were derived from the three dosage treatment groups and are presented in Table 1. The geometric mean serum concentrations (C_max_) of tulathromycin were 1.08, 3.49, and 7.39 μg/mL for the 1, 10, and 20 mg/kg doses, respectively, and were observed at 0.5 h after administration (Figure 1). The mean half-lives, T_1/2β_, were 25.7, 26.9, 28.6 h for the three respective doses. In addition, a concentration dependent AUC_168h_ was observed where mean AUC_168h_ increased with the increase in dosage (11.02, 57.18, and 89.60 μg·h /mL for 1, 10, and 20 mg/kg, respectively). For lung tissue, the mean values of PK parameters derived from the three treatment groups are presented in Table 2. With respect to each successive dosage level, the mean C_max_ was 0.15, 5.21, and 10.14 μg/g and t_max_ was 0.5, 0.5, and 1 h post-administration (Figure 2). The mean half-lives in lung tissue were about 67.4, 72.1, and 70.1 h for the three different dose treatments, respectively. Similar to the serum results, a concentration dependent AUC_168h_ was observed where mean AUC_168h_ increased with the increase in dosage (9.59, 262.78, and 434.95 μg·h /g for 1, 10, and 20 mg/kg, respectively).

**Table 1:** Pharmacokinetic parameters of tulathromycin in serum following single dose intramuscular administration at 1, 10, or 20 mg/kg in *H. parasuis* infected guinea pigs (mean ± SD, *n* = 8/group).

Parameters^*^	**Dose (mg/kg)**		
	**1**	**10**	**20**
T_1/2β_ (h)	25.7 ± 5.8	26.9 ± 3.8	28.9 ± 5.1
T_max_ (h)	0.5 ± 0.1	0.5 ± 0.1	0.5 ± 0.1
C_max_ (μg/ml)	1.08 ± 0.27	3.45 ± 4.53	7.39 ± 12.23
AUC_0−168*h*_ (μg·h/ml)	11.02 ± 2.38	57.18 ± 13.05	89.60 ± 19.89

* *T_1/2β_, elimination half-life; T_max_, time of maximum serum concentration; C_max_, maximum serum concentration; AUC_0-168h_, 168-h area under serum concentration-time curve*.

**Table 2:** Pharmacokinetic parameters of tulathromycin in lung tissues following single dose intramuscular administration at 1, 10, or 20 mg/kg in *H. parasuis* infected guinea pigs (mean ± SD, *n* = 8/group).

Parameters ^*^	**Dose (mg/kg)**		
	**1**	**10**	**20**
T_1/2β_ (h)	67.4 ± 8.5	72.1 ± 5.3	70.1 ± 11.6
T_max_ (h)	0.5 ± 0.2	0.5 ± 0.2	1 ± 0.4
C_max_ (μg/g)	0.15 ± 0.04	5.21 ± 0.64	10.14 ± 2.66
AUC_0−168*h*_ (μg·h/g)	9.59 ± 1.89	262.78 ± 57.09	434.95 ± 93.45

* *T_1/2β_, elimination half-life; T_max_, time of maximum serum concentration; C_max_, maximum serum concentration; AUC_0-168h_, 168-h area under serum concentration-time curve*.

Because AUC_168h_ linearly increased with dose from 1 to 20 mg/kg, we were able to interpolate values for AUC_168h_ at 2, 4, 6, 8, and 15 mg/kg. Of the serum samples, significant correlations between dose treatment and AUC_168h_ (*R*^2^ = 0.9829) or C_max_ (*R*^2^ =0.9889) were observed. Similarly for lung samples, significant correlations between dose treatment and AUC_168h_ (*R*^2^ =0.9806) or C_max_ (*R*^2^ =0.9986) were observed.

### *In vivo* Effectiveness of Tulathromycin in the Neutropenic Guinea Pig Intraperitoneal Infection Model

**Figure 3:**
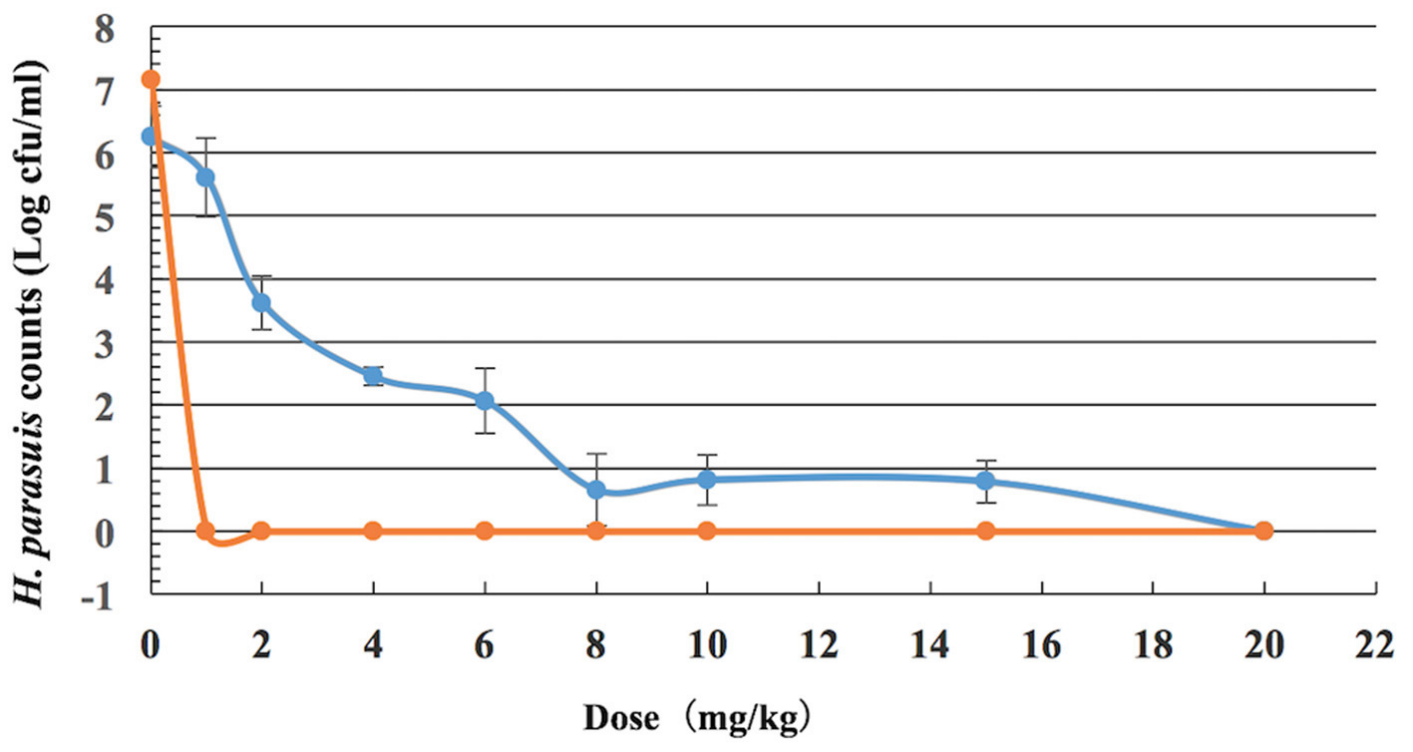
Viable counts of *H. parasuis* in blood and lung tissues of guinea pigs after a single dose of tulathromycin (*n* = 8/dose).

The viable counts of *H. parasuis* in lung tissues and blood from guinea pigs given doses of 0, 1, 2, 4, 6, 8, 10, 15, and 20 mg/kg of tulathromycin decreased with the increasing levels of dose, which implies that the bacteria loading decreased. In serums, the viable counts decreased sharply between dosages of 0 and 1 mg/kg. In lung tissues, the viable counts decreased sharply between dosages of 1 and 8 mg/kg, and then moderately varied between 8 and 20 mg/kg. The calculated number of *H. parasuis in vivo* after drug treatment is shown in Figure 3.

### Tulathromycin PK/PD Profiles

**Figure 4:**
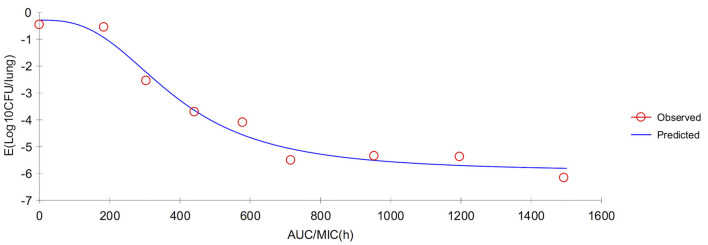
Sigmoid *E*_*max*_ relationship between anti-*Haemophilus parasuis* effect (*E*, log_10_ CFU/serum) and *in vivo* AUC_168_/MIC ratio against *H. parasuis* in the serum of guinea pigs.

In serum, the ratios of AUC_72h_/MIC and AUC_168h_/MIC for each dose of 0, 1, 2, 4, 6, 8, 10, 15, and 20 mg/kg were 0, 331.48, 435.78, 644.38, 852.98, 1,061.58, 1,270.18, 1,791.68, 2,313.18 h, and 0, 367.32, 607.95, 882.56, 1,157.17, 1,431.79, 1,906.08, 2,392.93, 2,986.61 h, respectively. The parameter AUC_72h_/MIC and AUC_168h_/MIC highly correlated with tulathromycin effectiveness (*R*^2^ = 0.9863 and 0.9878, respectively). The values of AUC_72h_/MIC of serum for *H. parasuis* 1-, 2-, 3- and 4-log_10_ CFU/mL reduction were 268.84, 399.99, 521.9, and 650.28 h, respectively (Table 3). The values of AUC_168h_/MIC of serum for *H. parasuis* 1-, 2-, 3-, and 4-log_10_ CFU/mL reduction were 366.06, 552.51, 728.47, and 916.90 h, respectively (Table 3). The profile of the sigmoid *E*_*max*_ model illustrating the relationship of antibiotic effectiveness and AUC_168h_/MIC is presented in Figure 4. The EC_50_ of AUC_72h_/MIC (h) and AUC_168h_/MIC were 544.21 and 761.04 h, respectively, and the slope of the graph of the AUC_72h_/MIC (h) and AUC_168h_/MIC ratio vs. effectiveness were 2.82 and 2.72, respectively (Table 3).

**Table 3:** Pharmacodynamic analysis of tulathromycin in a *H. parasuis* intraperitoneal infection model.

Parameter^*^	**AUC**_**0–72 h**_ **/MIC**		**AUC**_**0–168 h**_ **/MIC**	
	**Serum**	**Lung**	**Serum**	**Lung**
*E_*max*_* [log_10_ cfu/mL (or g)]	−0.31	−0.40	−0.31	−0.40
*E_0_* [log_10_ cfu/mL (or g)]	−6.03	−6.62	−6.03	−6.62
EC_50_	544.21	1,245.04	761.04	2,726.90
AUC/MIC (h) for 1-log_10_ cfu/mL (or g) reduction	268.84	235.31	366.06	507.20
AUC/MIC (h) for 2-log_10_ cfu/mL (or g) reduction	399.99	570.23	552.51	1,227.11
AUC/MIC (h) for 3-log_10_ cfu/mL (or g) reduction	521.90	992.30	728.47	2,126.44
AUC/MIC (h) for 4-log_10_ cfu/mL (or g) reduction	650.28	1,710.68	916.90	3,462.62
Slope (*N*)	2.82	1.21	2.72	1.33

* *E_0_ is the change in log_10_ cfu/mL in the control sample (absent of tulathromycin); E_max_ is the difference in effect between the greatest amount of growth (observed in the growth control, E_0_) and the greatest amount of mortality. EC_50_ is the AUC/MIC value producing a 50% reduction in bacterial counts and N is the Hill coefficient that describes the steepness of the AUC/MIC-effect curve*.

**Figure 5:**
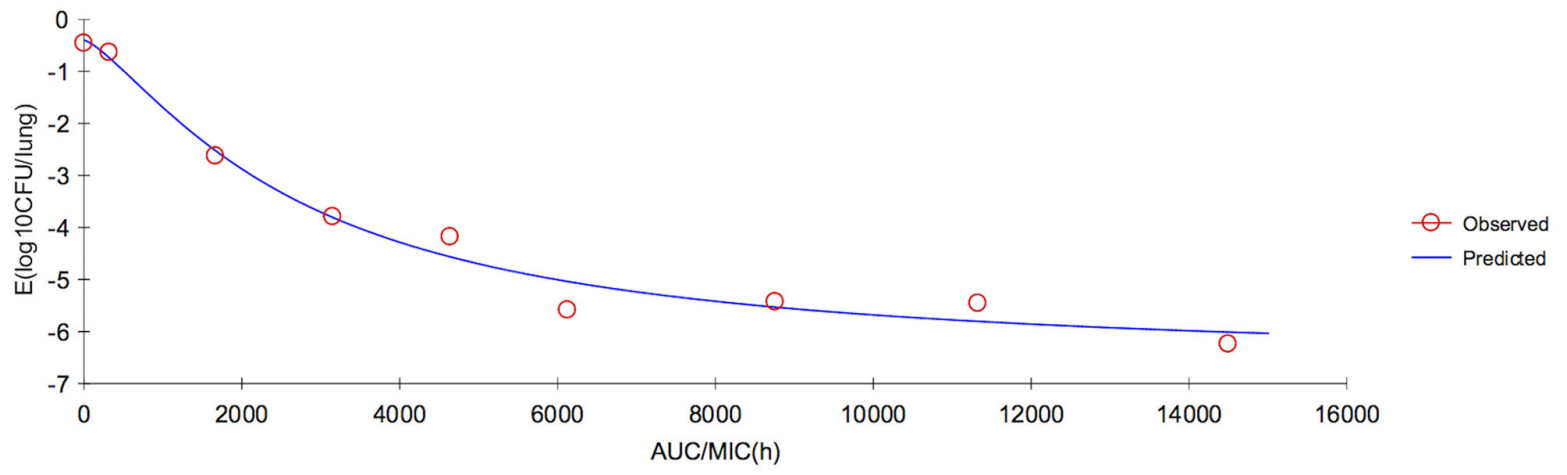
Sigmoid *E*_*max*_ relationship between anti-*Haemophilus parasuis* effect (*E*, log_10_ CFU/lung) and *in vivo* AUC_168_/MIC ratio against *H. parasuis* in the lung tissues of guinea pigs.

In lung tissue, the ratios of AUC_72h_/MIC and AUC_168h_/MIC for each dose of 0, 1, 2, 4, 6, 8, 10, 15, and 20 mg/kg were 0, 280.36, 742.16, 1,665.76, 2,589.36, 3,512.96, 4,436.56, 6,745.56, 9,054.56 h, and 0, 319.79, 1,667.00, 3,153.14, 4,639.27, 6,125.40, 8,759.35, 11,326.87, and 14,498.35 h, respectively. The parameter AUC_72h_/MIC and AUC_168h_/MIC highly correlated with antibiotic effectiveness (*R*^2^ = 0.9985 and 0.9911, respectively). The values of AUC_72h_/MIC of serum for *H. parasuis* 1-, 2-, 3-, and 4-log_10_ CFU/mL reduction were 235.31, 570.23, 992.3, and 1,710.68 h, respectively. The values of AUC_168h_/MIC (h) of lung tissue for *H. parasuis* 1-, 2-, 3-, and 4-log_10_ CFU/mL reduction were 507.20, 1,227.11, 2,126.44, and 3,462.62 h, respectively (Table 3). The profile of the sigmoid *E*_*max*_ model illustrating the relationship of antibiotic effectiveness and AUC_168h_/MIC (h) is presented in Figure 5. The EC_50_ of AUC_72h_/MIC (h) and AUC_168h_/MIC were 1,227.11 and 2,726.90 h, respectively, and the slope of the graph of the AUC_72h_/MIC (h) and AUC_168h_/MIC ratio vs. effectiveness were 1.21 and 1.33, respectively (Table 3).

### Dosage Calculation

The MIC of 13R was substituted for the MIC_90_ because we obtained an insufficient amount of tulathromycin MIC data to provide an estimate of MIC_90_ in serum. In this study, the single dosage values to achieve a reduction of 1-, 2-, 3-, and 4-log_10_ CFU/lung were calculated from the ratios of AUC_72h_/MIC were 3.8, 5.7, 7.5, and 9.3 mg/kg for serum and 1.7, 2.5, 4.3, and 7.4 mg/kg for lung tissue (Table 4), respectively. For AUC_168h_/MIC, the single dosage values to achieve a reduction of 1-, 2-, 3-, and 4-log_10_ CFU/lung were 8.7, 13.2, 17.4, and 21.9 mg/kg for serum and 3.7, 8.9, 15.4, and 25.1 mg/kg for lung tissue (Table 4), respectively.

**Table 4:** The single dosage values (mg/kg) calculated from the ratios of AUC/MIC against *H. parasuis*.

**Parameter**	**AUC**_**0–72 h**_ **/MIC**		**AUC**_**0–168 h**_ **/MIC**	
	**Serum**	**Lung**	**Serum**	**Lung**
AUC/MIC for 1-log_10_ cfu/mL (or g) reduction	3.8	1.7	8.7	3.7
AUC/MIC for 2-og_10_ cfu/mL (or g) reduction	5.7	2.5	13.2	8.9
AUC/MIC for 3-log_10_ cfu/mL (or g) reduction	7.5	4.3	17.4	15.4
AUC/MIC for 4-log_10_ cfu/mL (or g) reduction	9.3	7.4	21.9	25.1

## Discussion

*H. parasuis* is a common source of bacterial respiratory infections in pigs, such as Glasser's disease (57–60) which can be found in the majority of the lobes of lungs in animals with pneumonia (61–65). Tulathromycin has been widely used in more than 30 countries across America, Europe, Oceania, and Asia (66). Selection of antimicrobial-resistant bacteria is a constant risk with the application of antimicrobials (59). Thus, in order to obtain a dosage regimen to ensure clinical efficacy and minimize risk of selection of antibiotic resistance, an *in vivo* model was used to examine the PK/PD of tulathromycin in this study.

In some cases, it has been difficult to use PK/PD integration to assess the efficacy of some antimicrobial drugs, particularly drugs with long half-lives against respiratory bacteria. To the best of our knowledge, some authors have determined appropriate dosage regimens of other antibiotics, such as marbofloxacin (67, 68) and cefquinome (29) to eliminate *H. parasuis* infection. Other researchers have successfully predicted optimal doses of tulathromycin to eliminate other bacteria, such as *Streptococcus suis* (39), *Mannheimia haemolytica* (40), and *Pasteurela multocida* (38, 40) by *in vitro* PK/PD models. Untreated *H. parasuis* infection of the blood can lead to septicemia and serofibrinous serositis in various tissues (3, 4, 50). Thus, the threshold loads of *H. parasuis* in blood and lungs that can lead to these serious conditions should be determined to help avoid greater morbidity and mortality. From the results of our *in vivo* infection model, we ascertained the threshold loads of *H. parasuis* at (7.15 ± 0.49) log_10_ CFU/blood and (6.24 ± 0.62) log_10_ CFU/lung, when the inoculum size was 9.26 log_10_ CFU/mL *via* intraperitoneal injection. Therefore, it is optimal to apply a single inoculum of a 0.2 mL aliquot solution containing ~10^9^ CFU of the *H. parasuis* strain in an *in vivo H. parasuis* infection model. These findings show that a threshold load should be determined to avoid an infected animal from dying.

Currently, there is little information on *in vivo* infection models of macrolide antibacterial agents against *H. parasuis*. To the best of our knowledge, this study is the first to investigate the efficacy of tulathromycin against *H. parasuis* using a neutropenic guinea pig infection model in the veterinary field. The advantages of our *in vivo* model over *in vitro* models and the main innovative points of the overall research are as follows: (1) *H. parasuis* colonies in blood and lung tissues of guinea pigs were simultaneously quantitated for dose optimization; (2) The drug concentrations in plasma and lung tissue were concurrently detected for the *in vivo* PK/PD model. (3) This is the first time that the dose proportionality of tulathromycin PKs in the dose range of 1 to 20 mg/kg has been described in guinea pig based on AUC (in serum and lung) and MIC (in serum). (4) An experimental animal model can be used instead of a target animal model to predict dose regimen of tulathromycin based on an *in vivo* PK/PD model. Both guinea pigs and pigs infected with *H. parasuis* produce similar symptoms of systemic infection; however, because tulathromycin targets lungs (42, 44), laboratory animal models are more beneficial because they provide more lung tissue data.

In the present study, pharmacokinetics of tulathromycin were investigated by examining tissue infection response to single doses of 1, 10, and 20 mg/kg in an *H. parasuis* infection model. The PK profiles revealed that the average tulathromycin concentration and AUC in lung tissues were higher than the concentration and AUC in serum at the same sampling periods (0–168 h) which supports that tulathromycin targets lung tissues. For the doses of 10 and 20 mg/kg, the C_max_ values of tulathromycin for lung tissue were 5.21 and 10.14 μg/g, respectively, and for serum were 3.45 and 7.39 μg/ml, respectively. The C_max_ lung/serum ratios were 1.5:1 and 1.4:1, respectively. The lung AUCs (at the last time point of 7 days) were 262.79 and 434.95 μg·h/ml and the serum AUCs (at the last time point of 7 days) were 57.18 and 89.6 μg·h/ml with respect to the 10 and 20 mg/kg doses. The AUC lung/serum ratios were 4.6:1 and 4.8:1, respectively. These results were similar to previously published articles. For example, the C_max_ lung/serum ratio was 5.6:1 in pig (42), 8.0:1 in cattle (44), and 1.3:1 (7 mg/kg) or 2.4:1 (28 mg/kg) in mice (47). However, the lungs were not a reservoir for tulathromycin to be then gradually released into the blood. In fact, it is the other way around. Tulathromycin gradually accumulate in the lungs over several days. Nowakowski et al. reported the progressive increase of the tulathromycin concentration ratio between lung and plasma ranging from 15 (at 12 h post administration) to 220 (at 360 h post administration) (44), which seems to be the case in the present study. It means that the lungs act as a sink, not a reservoir.

The AUC lung/serum ratio was 51.3:1 in pig (42), 56.5:1 in cattle (44), and 5.7:1 (7 mg/kg) or 7.9:1 (28 mg/kg) in mice (47). However, at a dose of 1 mg/kg, the average tulathromycin concentration in lung tissue was lower than the corresponding concentration in serum between 5 min and 48 h after administration, which is similar to a report of a C_max_ lung/serum ratio of 0.6:1 in infected mice with a dose of 7 mg/kg (47). Research has shown that a total tissular concentration has no therapeutic meaning (69). This is particularly true for tulathromycin that accumulates massively in phagolysosome of the lung because their pH is very low (ionic trapping). This pH is can be as low as 4.5(70) and for this pH, tulathromycin loses all its activity (71, 72). It means it is not this high local lung concentration, which is responsible for the microbiological effects of tulathromycin on lung pathogens. In addition, for mild infections, *H. parasuis* is mainly localized in extracellular areas (73). These findings show that lab animal models can be used to closely represent target animals' PK/PDs.

Of the other PK parameters that we examined, T_max_ in serum (0.5 h) was similar to previous studies: 0.5 h in mice (47), 1 h in rabbits (48), 0.25 h in pig (42), and 1.8 h in cattle (44). In lung tissue, T_max_ ranged between 0.5 and 1 h in the present study and was similar to other laboratory animals' ranges: 0.5–1 h in mice (47) and 1 h in rabbits (48). However, our T_max_ was more early than that found in target animals: 24 h in pig (42) and 24 h in cattle (44). It was because the terminal half-life is shorter the above animals. In contrast, T_1/2β_ in serum ranged from 25.7 to 28.9 h in guinea pig, which was longer than that found in mice, 13.5–18.1 h (47), and shorter than that in rabbits, 31.69 h (48); pig, 75.6 h (42), and 78.7 h (74); and cattle, 90 h (44). In lung tissue, T_1/2β_ (a range of 67.4–72.1 h) in the present study was shorter than that of other animals, such as, a range of 82 h−112 h in mice (47), 142 h in pig (42), and 184 h in cattle (44). We speculate that the metabolism and distribution of tulathromycin in lungs may have been reduced by the swollen lesions that we observed in the tissues. Moreover, laboratory animal models, commonly used in studies, typically have much faster rates of elimination of antibiotics than target animals (75).

Antibiotic resistance and its development are a complex subject. Owing to the over-use of antibiotics and cross resistance, antibiotic resistance is on the rise and in particular, more bacteria are developing resistance against tulathromycin (76, 77). Thus, a suitable dose rate must be determined to ensure the continued efficacy of tulathromycin for susceptible organisms and to minimize selection for antibiotic resistance in bacteria (78, 79). In the present study, we found that we can estimate the AUC_168h_ for any dose level from 1–20 mg/kg based on the linear relationship between our treatment levels of dose (1, 10, 20 mg/kg) and AUC_168h_ (*R*^2^ = 0.9829 and *R*^2^ = 0.9806 for serum and lung tissue, respectively). These results indicate that this analytical chemistry method is a reasonable tool to derive meaningful PK profiles that may help predict tulathromycin efficacy. This relationship supports previous studies that examined tulathromycin PK in pig within a range of 0.94–4.77 mg/kg (43) and tulathromycin PK in cattle within a range of 1.27–5.05 mg/kg (43), where the *R*^2^ were 0.9982 and 0.9191, respectively. Altogether, these studies suggest that tulathromycin is characterized by linear pharmacokinetics both in lab and target animals.

The MIC of tulathromycin against *H. parasuis* in serum was tested, where the CAMHB /serum ratio was 16:1 (MIC_CAMHB_ = 0.5 ug/mL and MIC_serum_ = 0.03 ug/mL). Thus, tulathromycin was subjected to a large serum effect. Furthermore, ratios of MIC_broth_/MIC_biologicmatrix_ of tulathromycin against other bacteria have been reported in previous studies that are similar to the ratios determined in our study (38–40, 80). Our *in vivo* MIC results indicate that PKPD-based dose prediction of tulathromycin is promising for treatment of *H. parasuis* infection. Overall findings suggest that an *in vivo* rather than *in vitro* experiment may be the better method to determine tulathromycin efficacy and that pharmacodynamic data obtained in biological fluids is very important (80), and more suitable for PK/PD research (40).

Our data showed high correlation among the PK/PD index, AUC/MIC, and the *in vivo* antibacterial effects of tulathromycin against *H. parasuis* (*R*^2^ > 0.98 and *R*^2^ > 0.99 for serum and lung tissue, respectively), which supports previous studies of tulathromycin activity against *Streptococcus suis* (39) and *Pasteurella multocida* (38). Different doses of AUC/MIC (0–72 or 0–168 h) for serum and lung were shown in Table 5. In a recent review on PK/PD of antibiotics in veterinary medicine (81), it is reported in the section entitled *In vivo* infection models that the primary end-point is reduction in bacterial burden in the infected tissue, which is typically assessed at 24 h after initiation of AMD therapy. Bacteriostasis and 1- or 2-log_10_ reduction in count at 24 h are commonly used end-points, as they are correlated with limited or greater levels of clinical outcomes, respectively. In this study, to assess the different possible clinical situations ranging from a mild infection in a non-immunocompromised animal up to a severe infection in an immunocompromised animal, we compute AUC/MIC corresponding to 1-, 2-, 3-, and 4-log_10_ based on AUC_72h_/MIC and AUC_168h_/MIC, respectively. In fact, the values of the PK/PD indexes are routinely given in terms of free concentrations, not total concentration. In the previous study, the free fraction (fu) of tulathromycin in guinea pig serum was 0.56–0.74 (41). Thus, the PK/PD index, fAUC/MIC (0–72 h) corresponding to 1-, 2-, 3-, and 4-log_10_ were 174.75, 259.99, 339.24, 422.68 h, respectively, and fAUC/MIC (0–168 h) corresponding to 1-, 2-, 3-, and 4-log_10_ were 237.94, 359.13, 473.51, 595.99 h, respectively. Here our goal is only to calculate a dose and so we can do this directly from the total plasma concentrations. According to the calculation results, we found that the computed dose more lower using as objective a 2-log_10_ reduction and an AUC_0−72h_ as the value of the PK/PD index than AUC_0−168h_. In our previous research, which published in PLOS (41) showed that doses of 7.2–8.0 mg/kg of tulathromycin *via* an *in vitro* model resulted in high eradication rates (99.99%). In this study, the computed doses to achieve a reduction of 2-log_10_ CFU/lung from the ratios of AUC_72h_/MIC were 5.7 mg/kg for serum and 2.5 mg/kg for lung tissue, which lower than the values of AUC_168h_/MIC to achieve a reduction of 2-log_10_ CFU/lung were 13.2 mg/kg for serum and 8.9 mg/kg for lung tissue, respectively. Using as objective a 2-log_10_ reduction and an AUC (0–72 h) as the value of the PK/PD index, an equivalent dose for target animals can be calculated with a dose conversion coefficient, for example, the dose conversion coefficient for guinea pig to pig is 0.296 and results in a converted doses of 1.7 mg/kg (serum) and 0.7 mg/kg (lung) for pig (82). While, to obtain a reduction of 4-log_10_ CFU/ml (or g) in the lungs, the converted doses were 2.8 mg/kg (serum) and 2.2 mg/kg (lung) for pig (Table 4), which is similar to the recommended dose for SRD of 2.5 mg/kg (31, 71). Our calculated doses against *H. parasuis* were different from other studies which have determined a tulathromycin dosage of 13.25 mg/kg against *Pasteurella multocida* (38) and 3.56 mg/kg against *Streptococcus suis* (39). Both of these examples were also higher than the recommended dose (2.5 mg/kg). In contrast, results in this study were similar to the results of a previous study that reported an estimated dosage of 2.52 mg/kg of tulathromycin against *Pasteurella multocida* (40). The difference may be explained by the variable responses of different bacteria to tulathromycin which likely results in different PK/PD models.

**Table 5:** Different doses of AUC/MIC (0–72 h or 0–168 h) for serum and lung.

**Doses (mg/kg)**	**AUC**_**0–72h**_ **/MIC (h)**		**AUC**_**0–168 h**_ **/MIC (h)**	
	**Serum**	**Lung**	**Serum**	**Lung**
1	331.48	280.36	367.32	319.79
2	435.78	742.16	607.95	1,667.00
4	644.38	1,665.76	882.56	3,153.14
6	852.98	2,589.36	1,157.17	4,639.27
8	1,061.58	3,512.96	1,431.79	6,125.40
10	1,270.18	4,436.56	1,906.08	8,759.35
15	1,791.68	6,745.56	2,392.93	11,326.87
20	2,313.18	9,054.56	2,986.61	14,498.35

There are two limitations to our study. First, we assumed a value of 100% bioavailability for our dosage calculations because the reported bioavailabilities of tulathromycin are near 100% in other animals, such as 110% in pigs (42), 93.6% in cattle (44), 95.8% in goats (46), and 94.25% in rabbits (48). Second, we did not conduct a dose confirmation on a target animal. Thus, a follow-up study should include more *H. parasuis* strains to calculate MIC_90_ and the dose regimen should be validated in clinical practice on swine.

In conclusion, the present study characterized the *in vivo* effectiveness of tulathromycin against *H. parasuis* in a neutropenic guinea pig model. This infection model is similar to clinical infection or systemic infection in swine because it has the same clinical symptoms and pathological changes when infected by *H. parasuis*. Our study indicates that lung pharmacokinetics have an important role as a surrogate marker in establishing PK/PD relationships and clinical efficacy against bacterial respiratory infections, especially for macrolide antibiotics. Furthmore, the *in vivo* data suggest that animal dosage regimens more accurate than data from *in vitro* model. Lastly, the computed dose could be more realistic using as objective a 2-log_10_ reduction and an AUC (0–72 h) as the value of the PK/PD index.

## Data Availability Statement

The original contributions presented in the study are included in the article/Supplementary Material, further inquiries can be directed to the corresponding author/s.

## Ethics Statement

The animal study was reviewed and approved by Qingdao Agriculture University Animal Ethics Committee approved the animal experiment procedures (No. 2020-025).

## Author Contributions

Y-dZ and B-hF conceived and designed the experiment. L-hW, S-jL, and L-lG performed the experiments. L-lG and R-yG analyzed data and drafted the manuscript. All authors read and approved the final manuscript.

## Funding

This work was supported by the Development of new raw materials and preparations for Animal Respiratory Disease Control under Grant 20201220 and the PhD Fund of Qingdao Agricultural University (663-1119017), and Shandong Provincial College Student Innovation and Entrepreneurship Training Program Project (S202010435088).

## Conflict of Interest

The authors declare that the research was conducted in the absence of any commercial or financial relationships that could be construed as a potential conflict of interest.

## Publisher's Note

All claims expressed in this article are solely those of the authors and do not necessarily represent those of their affiliated organizations, or those of the publisher, the editors and the reviewers. Any product that may be evaluated in this article, or claim that may be made by its manufacturer, is not guaranteed or endorsed by the publisher.
